# Extraction and Evaluation of Discriminative Indexes of the Wearing Condition for High-Precision Blood Pressure Pulse Wave Measurement

**DOI:** 10.3390/mi13050679

**Published:** 2022-04-27

**Authors:** Yosuke Osawa, Tetsuji Dohi

**Affiliations:** Micro System Laboratory, Chuo University, 1-13-27, Kasuga, Bunkyo-ku, Tokyo 112-8551, Japan

**Keywords:** MEMS 3-axis sensor, arterial tonometry, blood pressure pulse wave

## Abstract

In this paper, we report a new discriminant index for evaluating the wearing condition of a blood pressure pulse wave measurement device with small variability. The prototype device consists of MEMS 3-axis force sensors, a 3-axis push-in adjustment mechanism, a fixed jig, and a signal processing board. The arterial tonometry method is used by this device to measure blood pressure pulse waves. However, to accurately measure the blood pressure pulse wave, the device must be appropriately worn. The evaluation of the wearing condition using the amplitude of the blood pressure pulse wave was complicated by the wearing process due to the large variability. Therefore, we propose the ratio of the blood pressure pulse wave component to the noise component as an index that can evaluate the wear condition of the device. The correlation coefficient between the discriminant index and the amplitude of the blood pressure pulse wave had a correlation coefficient of 0.879. Furthermore, the discriminant index reduced the coefficient of variation by 12.8%. Therefore, it was suggested that the discriminant index is an index that can evaluate the pressing amount, one of the wearing conditions of the blood pressure pulse wave measurement device with a small influence of variation.

## 1. Introduction

Hypertension, in which high blood pressure continues, is said to accelerate arteriosclerosis and is said to be a cause of the increased risk of myocardial infarction and stroke [1]. In addition, blood pressure changes depending on one’s state and environment such as during activity and rest and fluctuation during the day are also significant. Sudden changes in blood pressure, especially, may cause sudden changes in symptoms in cardiovascular disorders such as myocardial infarction and stroke [2]. As a result, it is important to detect these risks in advance by monitoring the rapid changes in blood pressure through continuous blood pressure measurement at each beat during daily life.

**Figure 1:**
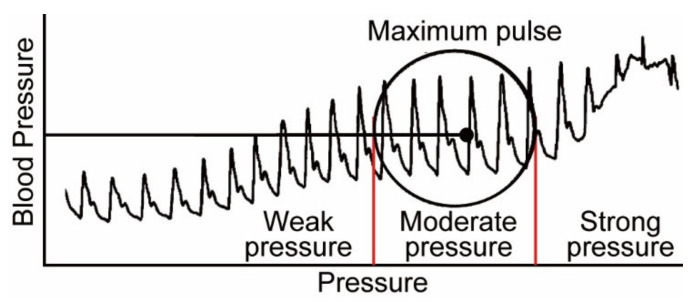
Changes in the amplitude of the blood pressure pulse wave when the wearing state is changed [9].

**Figure 2:**
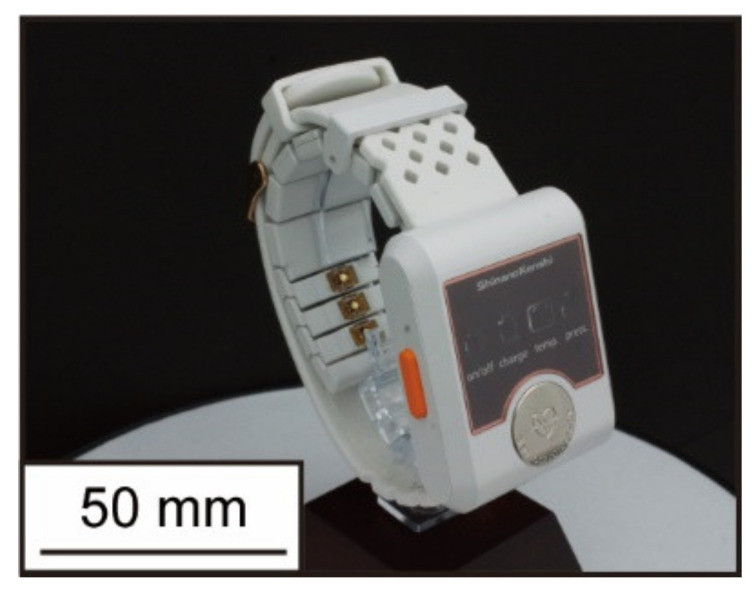
Wristwatch-type device being developed in our lab.

The oscillometric method is one of the most commonly used methods for measuring blood pressure in daily life. In this measurement method, the upper arm is pressurized by the air pressure of a cuff wrapped around the arm, and the systolic and diastolic pressures are measured by the pressure change during the decompression process [3]. Using this principle, there is Ambulatory Blood Pressure Monitoring (ABPM), which can measure blood pressure at a high frequency every 30 min. This device is not only unable to measure continuous blood pressure with each beat, but it has also been reported to cause acute neuralgia and sleep disturbance due to the high-pressure load [4,5], whereas arterial tonometry is one of the methods that can measure blood pressure continuously and with low load [6,7]. This method enables continuous and low-load blood pressure measurement as if palpated by a force sensor placed directly above the radial artery. On the other hand, to accurately measure blood pressure by this method, the device must be worn in the proper condition [8]. The following parameters are used to evaluate the wearing condition of the device: the position of the sensor and the radial artery, the angle between the wrist and the sensor surface, and the amount of pressure on the sensor. Dueck and others showed good agreement between invasive and noninvasive radial artery blood pressures when the device was worn, such that the amplitude of the blood pressure pulse wave was maximized [9]. The amplitude of the blood pressure pulse wave increased with the sensor pressing force in this study, as shown in Figure 1, and the maximum amplitude value was used as an index to evaluate the wear condition of the arterial tonometry method. Kato and others developed a device with pressure sensor arrays that can be worn automatically with maximum amplitude using air pressure [10]. Furthermore, Canning and others developed a headphone-type sphygmomanometer and achieved blood pressure pulse wave measurement at the superficial temporal artery [11]. This device uses a stepping motor to automatically adjust the amount of pressure on the sensor so that measurements can be made according to the appropriate mounting condition. Although these devices can automatically adjust the wearing condition, creating wearable devices with motors and pneumatics is difficult because the devices are heavy and consume large amounts of electricity. In contrast, in our laboratory, we have been developing a wristwatch-type wearable device that is small, lightweight, and capable of continuously measuring blood pressure pulse waves [12,13,14]. A photograph of the latest prototype device is shown in Figure 2. While this device is small, lightweight, and can continuously measure blood pressure pulse waves, the wearing process is complicated and time-consuming for high-precision blood pressure measurement. Specifically, it is necessary to confirm that the amplitude of the blood pressure pulse wave is maximum at each of the following: the position of the sensor and radial artery, the angle between the wrist and sensor surface, and the amount of sensor pressure. Additionally, because the amplitude of the blood pressure pulse wave varies from measurement to measurement and has large variability, it is necessary to confirm it at each wear. Therefore, a discrimination index of the device’s wearing state with small variability is required to make the evaluation of wear easier.

In this study, we developed a prototype device using the arterial tonometry method and measured blood pressure pulse waves while changing the wearing condition. Furthermore, the measured waveform is frequency analyzed to determine the discriminant index related to the wearing condition of the device. The purpose of this study is to confirm whether the discriminant index can evaluate the wearing condition and if it is a small variation index.

## 2. Principle and Device

**Figure 3:**
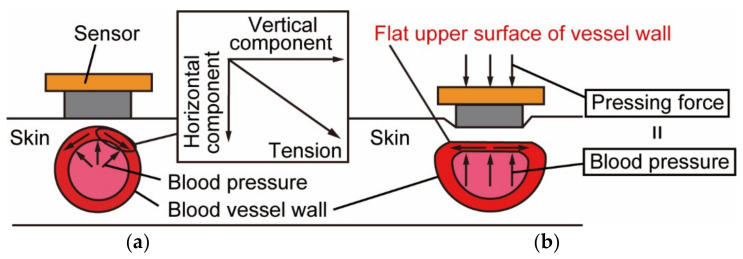
The principle of the arterial tonometry method: (**a**) Without pushing force; (**b**) With moderate pushing force.

Figure 3 shows the principle of the tonometry method, which enables continuous blood pressure measurement for each beat [15]. The device is mounted so that the MEMS 3-axis force sensor is placed directly above the radial artery, as shown in Figure 3a. When the sensor is not pressed against the skin surface, the measured force differs from blood pressure because it includes the vertical component of the tension acting in the circumferential direction of the vessel wall. As shown in Figure 3b, we flatten the upper part of the vessel by pressing the force sensor vertically against the skin just above the vessel with moderate pressing force. The vertical component of the tension acting on the vessel wall is reduced to zero in this way, and the pressing force and blood pressure are balanced. Therefore, the force sensor can directly measure blood pressure. Unlike the oscillometric method, which is a common blood pressure measurement method, the arterial tonometry method can measure blood pressure in a minimally invasive and continuous manner, since the measurement method does not block blood flow.

**Figure 4:**
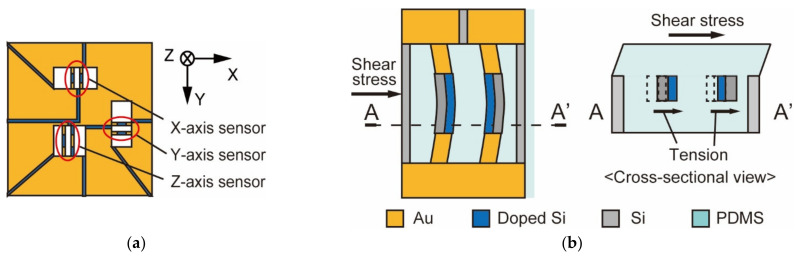
MEMS 3-axis force sensor: (**a**) Overview of the sensor; (**b**) The principle of the sensor.

The principle of the MEMS 3-axis force sensor used in this study is shown in Figure 4 [16]. The sensor shown in Figure 4a consists of three pairs of Si sidewall beams, one of which can measure vertical force and the other two can measure shear force. As an example, we will explain the principle of shear force measurement in this section. The beams formed in the sensor are arranged vertically, and the piezo-resistive layer is placed inside their sides. Since the MEMS 3-axis force sensor is embedded in the silicon rubber, the beam deforms in the direction of the force when the shear force is applied, as shown in Figure 4b. The resistance regions formed inside the two pairs expand and contract in the direction of deformation as the beams deform. The Wheatstone bridge circuit and the amplifier circuit detect the change in resistance due to the deformation of the beam, which is detected as a voltage change, and the shear force can be measured.

**Figure 5:**
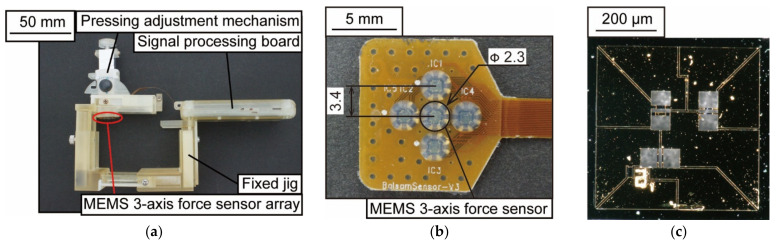
A photograph of the blood pressure pulse wave measurement device: (**a**) Wearable device; (**b**) MEMS 3-axis force sensor array; (**c**) Force sensor chip.

The appearance of the prototype device is shown in Figure 5. As shown in Figure 5a, the dimensions of the device are 95 × 75 × 45 mm^3^. The prototype device consists of a MEMS 3-axis force sensor array, a fixed jig, a 3-axis push-in adjustment mechanism, and a signal processing board. The MEMS 3-axis force sensor array shown in Figure 5b was developed in collaboration with Shinano Kenshi Co., Ltd. (Ueda, Japan) and Touchence Co. (Tokyo, Japan), and consists of five force sensors arranged in a crisscross pattern. The sensor element is a sensor chip embedded with PDMS, and the distance between the sensors is 3.4 mm. Figure 5c shows a photograph of the sensor chip. The dimensions of the sensor chip are 2.0 × 2.0 × 0.3 mm^3^, and the sampling frequency is 100 Hz. The fixed jig was fabricated using a 3D printer and can restrain the wrist. The 3-axis push-in adjustment mechanism can adjust the position and angle of the radial artery and sensor.

## 3. Experiments and Results

**Figure 6:**
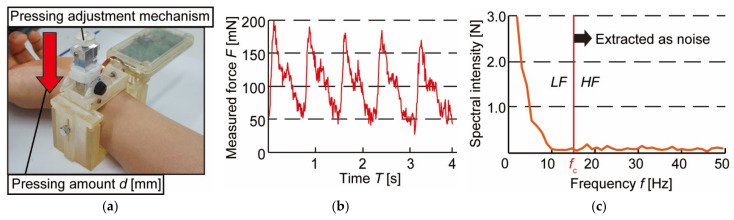
Experimental conditions and results of blood pressure pulse wave measurement: (**a**) Experimental setup; (**b**) Waveform of blood pressure pulse wave; (**c**) Fourier transformed blood pressure pulse wave.

We experimented to measure blood pressure pulse waves in the resting sitting position to confirm that the prototype can measure blood pressure. Figure 6a shows a photograph of the device, which was worn on the left wrist during the experiment. A healthy 25-year-old male subject participated in the measurement. The measurement waveform is shown in Figure 6b. The vertical axis represents the measured force *F* [mN], and the horizontal axis represents the measurement time *T* [s]. Local maxima and local minima could be confirmed periodically from the measurement form, and a single beat of the blood pressure pulse wave was extracted. The results of the Fourier transform of the extracted waveforms are shown in Figure 6c. It can be seen that the spectral intensity of the blood pressure pulse wave is quite low at *f* > 20 Hz. It is also known that at 0 < *f* ≤ 2 Hz, the heart rate is greatly affected by the main elements of the heart rate [17]. Therefore, the cut-off frequency *f*_c_ is determined between 2 < *f* ≤ 20 Hz. Then, the value of the integral for 2 < *f* ≤ *f*_c_ is defined as the low-frequency component (*LF*), and the value of the integral for *f* > *f*_c_ is defined as the high-frequency component (*HF*). The ratio of *LF* to *HF* (*LF*/*HF*) is proposed as an index to discriminate the wearing condition of the device.

Next, to confirm that the proposed discriminant index *LF*/*HF* (*f*_c_) is related to the device wearing state, blood pressure pulse wave measurement is conducted while varying the pressing amount of the sensor. The wearing condition of the device includes the pressing amount of the sensor, its position, and the sensor surface angle. In this experiment, the position of the sensor and the angle of the sensor surface were adjusted so that the amplitude of the pulse wave was always at its maximum. In this state, the pressing amount *d* [mm] shown in Figure 6a was increased by 1 mm every 20 s. The initial value of the pressing amount *d* was set at the position where the sensor made contact with the skin, and the experiment was conducted until the amplitude of the blood pressure pulse wave attenuated and disappeared.

**Figure 7:**
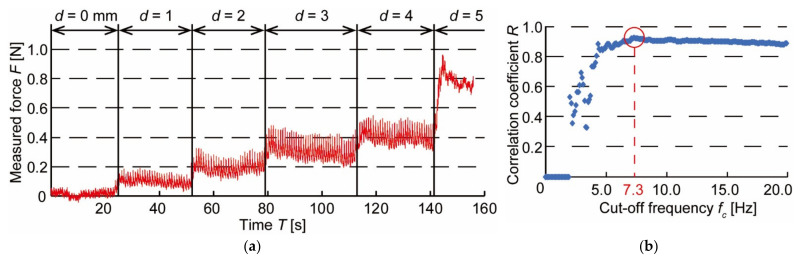
Results of pushing force change experiment: (**a**) Waveform of blood pressure pulse wave when pushing force is varied; (**b**) Changes in correlation coefficient between the amplitude of blood pressure *A*_P_ and discriminant index *LF*/*HF* when cut-off frequency *f*_c_ is varied.

The measurement waveform is shown in Figure 7a. The vertical axis represents the measured force *F* [N], and the horizontal axis represents the measurement time *T* [s]. In this experiment, the blood pressure pulse wave for each beat was extracted from the measured waveform and Fourier transformed as in the previous experiment. The discrimination indices *LF*/*HF* were calculated by varying the cut-off frequency *f*_c_. We then confirmed the relationship between *LF*/*HF*, and the amplitude of the blood pressure pulse wave, *A*_P_, which can evaluate the wearing state of the device. The correlation coefficients between *LF*/*HF* and *A*_P_ are shown in Figure 7b when *f*_c_ is varied. The correlation coefficient was maximum at *f*_c_ = 7.3 Hz and showed a very strong correlation of *R* = 0.924. This suggests that it is possible to discriminate against the wearing state of a device by *LF*/*HF*.

**Figure 8:**
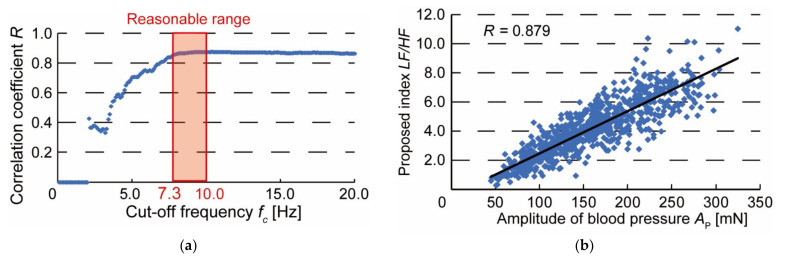
Results of repeated experiments: (**a**) Changes in correlation coefficient between the amplitude of blood pressure *A*_P_ and discriminant index *LF*/*HF* when cut-off frequency *f*_c_ is varied in repeated experiments; (**b**) Relationship between amplitude of blood pressure *A*_P_ and discriminant index *LF*/*HF* when *f*_c_ = 7.3 Hz.

Finally, to confirm the variability of the discrimination index *LF*/*HF*, we conducted 20 repetition experiments of changing the wearing condition. When the cut-off frequency *f*_c_ is varied, the correlation coefficient between *LF*/*HF* and the amplitude of the blood pressure pulse wave *A*_P_ is shown in Figure 8a. The results of the 20 experiments also show that the correlation coefficient begins to stabilize when *f*_c_ = 7.3 Hz. As shown in Figure 8b, the correlation coefficient between *A*_P_ and LF/HF was 0.879, indicating a strong correlation. When *f*_c_ = 7.3 Hz, Figure 8b is obtained from 20 experimental data. In addition, we compared the variability of *A*_P_ and *LF*/*HF* obtained when the amplitude of the blood pressure pulse wave was at its maximum in 20 experiments. The mean values, standard deviations, and coefficients of variation in the amplitude *A*_P_ and the index *LF*/*HF* of the blood pressure pulse wave are shown in Table 1. The coefficients of variation for *A*_P_ and *LF*/*HF* were 0.210 and 0.183, respectively, and we confirmed that the effect of variability was 12.8% lower for *LF*/*HF*. Therefore, by using the ratio of the low-frequency component *LF* to the high-frequency component *HF* of the blood pressure pulse wave, it is possible to discriminate the wearing condition of the blood pressure pulse wave measurement device with small variation.

## 4. Discussion

In Figure 6c, the blood pressure pulse wave was frequency analyzed by Fourier transform. When *f* > 12 Hz, the spectral intensity of the blood pressure pulse wave almost disappears. This is because the main frequency component of the blood pressure pulse wave is below 12 Hz [18] and the frequency component of the blood pressure pulse wave measured in this experiment is considered to be similar to that reported.

In Figure 7b, the cut-off frequency *f*_c_ at which the correlation coefficient between the amplitude of the blood pressure pulse wave, *A*_P_, and the proposed index, *LF*/*HF*, reached its maximum was 7.3 Hz. Indicating that the low-frequency component, *LF*, is the main frequency component of the blood pressure pulse wave. Since *f*_c_ is less than 12 Hz, it can be seen that the low-frequency component *LF* was extracted from the frequency component of the main blood pressure pulse wave. Therefore, *LF* is the blood pressure pulse wave component with a strong correlation with *A*_P_, while the high-frequency component *HF* is the blood pressure pulse wave component with little relationship to *A*_P_. In other words, the discriminant index *LF*/*HF* indicates the relative magnitude of the pulse wave component with the device wearing condition. Compared with *A*_P_, the low-frequency component *LF* is thought to accumulate features of the blood pressure pulse wave that are related to the degree of arterial stiffness and atrial contraction, including *A*_P_ [19,20]. Observation of these features is difficult when the device is not approximately worn, and the components are buried in noise. Therefore, there was a strong correlation between *A*_P_ and *LF*/*HF*, which is the ratio of waveform components and noise related to the wearing state. On the other hand, if the heart rate differs depending on the subject’s condition (e.g., after exercise) or individual differences, and if the volume of blood pushed out by the heart per unit time changes, the cut-off frequency fc is considered to be affected. In such cases, the optimal cut-off frequency in Figure 7b will change.

Figure 8b shows the correlation between the amplitude of the pulse wave, *A*_P_, and the discriminant index, *LF*/*HF* when the pressing force experiment was conducted 20 times. Although the optimal cut-off frequency fc varied from experiment to experiment, the correlation coefficient was calculated by fixing *f*_c_ at 7.3 Hz. However, the cut-off frequency must be determined for each subject because it may vary with heart rate and respiration rate. If the heart rate is different from normal, such as during exercise, the heart rate should be calmed down until it reaches a resting state. In addition, the coefficient of variation calculated in Table 1 tends to increase with the value of *f*_c_. Therefore, in this experiment, the effect of variation can be reduced by determining *f*_c_ in the range of 7.3–10 Hz, as shown in Figure 8a. It was no longer necessary to confirm that the discriminant index was at its maximum, and the time required to properly attach the device was greatly reduced. We suggest that this is a discriminant index of the wearing condition of a wearable device for easy and accurate blood pressure measurement.

## 5. Conclusions

In this study, we developed a prototype of a pulse wave measurement device that can be worn in different ways and measured the pulse wave using the arterial tonometry method. The measured waveform was analyzed by frequency and decomposed into high-frequency components *HF* and low-frequency components *LF*. We defined *LF*/*HF* as a new discriminant index, and the amplitude of the blood pressure pulse wave used to evaluate the wearing condition was compared. When the cut-off frequency was set at 7.3 Hz, the correlation coefficient between the amplitude of the blood pressure pulse wave and the discrimination index was *R* = 0.879. The coefficient of variation was reduced by 12.8% in the discriminant index compared to the amplitude of the blood pressure pulse wave. Therefore, the discriminant index is an index that can evaluate the wearing condition of the blood pressure pulse wave measurement device with a small influence of variation.

## Figures and Tables

**Table 1:** Comparison of the variability between the amplitude of blood pressure *A*_P_ and discriminant index *LF*/*HF*.

	Amplitude of Blood Pressure Pulse Wave *A*_P_ [mN]	Discriminant Index *LF*/*HF*
Average	208	5.74
Standard deviation	43.7	1.05
Coefficient of variation	0.210	0.183
